# Association between Lung Function in Adults and Plasma DDT and DDE Levels: Results from the Canadian Health Measures Survey

**DOI:** 10.1289/ehp.1408217

**Published:** 2014-12-23

**Authors:** Ming Ye, Jeremy Beach, Jonathan W. Martin, Ambikaipakan Senthilselvan

**Affiliations:** 1 School of Public Health; 2 Division of Preventive Medicine, Department of Medicine, and; 3 Division of Analytical and Environmental Toxicology, Department of Laboratory Medicine and Pathology, University of Alberta, Edmonton, Alberta, Canada

## Abstract

**Background:**

Although DDT [1,1,1-trichloro-2,2-bis(4-chlorophenyl)ethane] has been banned in many countries since the 1970s, it may still pose a risk to human respiratory health. In agriculture, DDT exposures have been associated with asthma and chronic bronchitis. However, little is known about the effect of DDT on lung function.

**Methods:**

We used data on 1,696 participants 20–79 years of age from the Canadian Health Measures Survey (CHMS) and conducted multiple regression analysis to estimate associations between plasma *p,p´*-DDT/DDE and lung function.

**Results:**

Almost all participants (> 99.0%) had detectable concentrations of plasma *p,p´*-DDE, but only 10.0% had detectable *p,p´*-DDT. Participants with detectable *p,p´*-DDT had significantly lower mean FVC (difference = 311 mL; 95% CI: –492, –130; *p* = 0.003) and FEV_1_ (difference = 232 mL; 95% CI: –408, –55; *p* = 0.015) than those without. A 100-ng/g lipid increase in plasma *p,p´*-DDE was associated with an 18.8-mL decrease in mean FVC (95% CI: –29, –9) and an 11.8-mL decrease in mean FEV_1_ (95% CI: –21, –3). Neither exposure was associated with FEV_1_/FVC ratio or FEF_25%–75%_.

**Conclusions:**

DDT exposures, which may have occurred decades ago, were still detectable among Canadians. Plasma DDT and DDE were negatively associated with lung function parameters. Additional research on the potential effects of DDT use on lung function is warranted.

**Citation:**

Ye M, Beach J, Martin JW, Senthilselvan A. 2015. Association between lung function in adults and plasma DDT and DDE levels: results from the Canadian Health Measures Survey. Environ Health Perspect 123:422–427; http://dx.doi.org/10.1289/ehp.1408217

Objectives: We examined DDT/DDE [1,1-bis-(4-chlorophenyl)-2,2-dichloroethene] concentrations in plasma and associations with lung function FVC (forced vital capacity), FEV_1_ (forced expiratory volume in 1 sec), FEV_1_/FVC ratio, and FEF_25%–75%_ (forced expiratory flow between 25% and 75% of FVC).

## Introduction

DDT [1,1,1-trichloro-2,2-bis(4-chlorophenyl)ethane], an organochlorine insecticide, was once widely used to control insects in agriculture (International Programme on Chemical Safety 1979) as well as insect-transmitted diseases, such as malaria and typhus (Attaran and Maharaj 2000). DDT can naturally break down into DDE [1,1-bis-(4-chlorophenyl)-2,2-dichloroethene] and DDD [1,1-dichloro-2,2-bis(*p*-chlorophenyl)ethane] through photolysis and microbial biodegradation [Agency for Toxic Substances and Disease Registry (ATSDR) 2002]. In humans, DDT can be either oxidized or reduced by cytochrome P450 enzymes (CYP450) to form DDE or DDD (Chen et al. 2009). DDE can further undergo epoxidation and phase 2 metabolism, and DDD can be further oxidized to DDA [2,2-bis(4-chlorophenyl)acetic acid] (Chen et al. 2009).

Both DDT and its breakdown products DDE and DDD are highly persistent in the environment. In the soil, DDT, DDE, and DDD can persist ≥ 40 years (ATSDR 2002). In addition, DDT and its breakdown products are highly lipophilic and have the potential to bioaccumulate in the fat tissue of exposed animals (Anderson 1985). DDT and its breakdown compounds can enter the human body through contaminated water, soil, and food (Bayen et al. 2005; Pérez-Maldonado et al. 2010). In humans, DDT and DDE have half-lives of 6 years and up to 10 years, respectively (Longnecker 2005; Wolff et al. 2000). Previous studies have shown that DDT and/or DDE were detectable in almost all human blood and breast milk samples, which were collected mainly in the 1990s and 2010s from a number of global regions (Eskenazi et al. 2009; Pérez-Maldonado et al. 2010; Smith 1999).

As a result of such environmental concerns, the use of DDT was greatly restricted or banned in most developed countries, including the United States, Canada, and many European countries, in the 1970s. A worldwide ban of DDT for agricultural use began in 2004 after the Stockholm Convention classified DDT as a persistent organic pollutant (POP) [United Nations Environment Programme (UNEP) 2010]. Nevertheless, because of its ongoing use for disease vector control in some countries, high environmental persistence, and bioaccumulative properties, DDT and its breakdown compounds still pose potential risks to human health. Many adverse effects on human health DDT exposures have been associated with a variety of outcomes, including neurological (Keifer and Firestone 2007), immunological (Corsini et al. 2008), reproductive (Beard and Australian Rural Health Research Collaboration 2006), and respiratory outcomes (Ye et al. 2013) and some cancers (Beard and Australian Rural Health Research Collaboration 2006). In addition, there is experimental evidence that DDT has endocrine-disrupting effects (De Coster and van Larebeke 2012).

A number of associations between respiratory health outcomes and DDT have been reported among agricultural pesticide applicators. For example, results from the U.S. Agricultural Health Study demonstrated that adult-onset asthma was associated with exposures to DDT among farmers (Hoppin et al. 2008, 2009). The authors further suggested associations appeared to be more specific for atopic asthma among women (Hoppin et al. 2008). Another report based on the Agricultural Health Study suggested that duration of DDT exposure was significantly associated with chronic bronchitis (Hoppin et al. 2007). A retrospective cohort study of outdoor pesticide applicators in Australia also reported that asthma mortality was higher among workers who were occupationally exposed to insecticides, including DDT (Beard et al. 2003).

Although there have been some studies of the effects of DDT exposure on respiratory diseases, few have focused on its impact on lung function. In the present study, we estimated the association of DDT and its metabolite DDE with lung function using data from the Canadian Health Measures Survey (CHMS).

## Methods

*Study population*. From 2007 through 2009, Statistics Canada conducted the Canadian Health Measures Survey (CHMS, cycle 1), a cross-sectional survey collecting baseline health information of Canadians (Statistics Canada 2011). In the present study, we used data on 1,696 participants 20–79 years of age from cycle 1 CHMS.

CHMS participants were chosen using a multistage sampling strategy, which included stratification of collection sites by geographic regions and census metropolitan areas (CMAs), selection of collection sites according to population size, sampling of dwellings within collection sites with stratification of dwellings by age groups of inhabitants, and sampling of individuals from dwellings in each age stratum. Next, a random selection of respondents provided the fasting blood samples in the age group 20–79 years (Statistics Canada 2011). People who were living on reserves and other Aboriginal settlements, residents of institutions, full-time members of the Canadian Forces, and those living in certain remote areas with low population densities were excluded from the CHMS (Statistics Canada 2011). A detailed description of the CHMS is available elsewhere (Statistics Canada 2011).

According to Statistics Canada (2011), the total survey population of cycle 1 CHMS (2007–2009) included 5,604 individuals from 15 collection sites in five Canadian provinces (New Brunswick, Quebec, Ontario, Alberta, and British Columbia), and was considered representative of 96.3% of the Canadian population. A subgroup of 1,696 individuals provided fasting blood samples for DDT and DDE measurement. Participation in CHMS was voluntary, and all 1,696 participants provided informed consent before participation, including consent for the storage and use of their blood samples for future studies (Statistics Canada 2011).

*Lung function measures*. Lung function parameters considered in this study were FVC (forced vital capacity), FEV_1_ (forced expiratory volume in 1 sec), FEV_1_/FVC ratio, and FEF_25%–75%_ (forced expiratory flow between 25% and 75% of FVC). Health measurement specialists measured lung function among the CHMS participants using a portable spirometer (Koko®; PDS Instrumentation Inc., Louisville, CO, USA). Calibration was performed using a 3-L syringe. Results were standardized to body temperature, barometric pressure, and water saturation (BTPS) (Statistics Canada 2011). American Thoracic Society (ATS) recommendations for performance of spirometry were followed, including obtaining a minimum of three acceptable trials from up to eight attempts based on the ATS definition of within- and between-maneuver criteria for usable and acceptable trials (Hendrick et al. 2002; Statistics Canada 2011). The largest value of FVC (or FEV_1_) from acceptable trials was used for measuring FVC (or FEV_1_) (Statistics Canada 2010a, 2011). The mean flow rate (milliters per second) of FEF_25%–75%_ from the acceptable trial with the largest sum of FVC and FEV_1_ was collected for measuring FEF_25%–75%_ (Statistics Canada 2010a, 2011).

p,p*´-DDT and* p,p*´-DDE concentrations in plasma*. In the present study, concentrations of *p,p´*-DDT and its major metabolite *p,p´*-DDE were measured in blood plasma. All blood samples were centrifuged within 2 hr and aliquoted within 4 hr after the blood was drawn (Statistics Canada 2011). Blood samples were then stored frozen at –20°C until concentrations of *p,p´*-DDT and *p,p´*-DDE were measured. Concentrations of *p,p´*-DDT and *p,p´*-DDE in blood plasma (micrograms per liter) were measured using gas chromatography–mass spectrometry (Health Canada 2010; Statistics Canada 2011). Detailed laboratory standard operating procedures are described at the Laboratoire de toxicologie, Institut national de santé publique du Québec (INSPQ) website (INSPQ 2009). Limits of detection (LOD) for *p,p´*-DDT and *p,p´*-DDE were 0.05 μg/L plasma and 0.09 μg/L plasma, respectively (Health Canada 2010). Concentrations of *p,p´*-DDT or *p,p´*-DDE (micrograms per liter plasma) were normalized to total blood lipids and converted to nanograms per gram lipid (Aylward et al. 2010; Health Canada 2010), with total blood lipids calculated as: total lipids (grams per liter) = 2.27 × 386.65 × cholesterol (moles per liter) + 885.45 × triglycerides (moles per liter) + 0.623, where 386.65 and 855.45 are the average molecular weights (grams per mole) of cholesterol and triglycerides, respectively (Health Canada 2010).

*Factors related to lung function*. We considered several factors that may affect lung function as potential confounders in our analyses, including demographic factors (age, sex, ethnicity, and immigration status), anthropomorphic data (standing height, weight), physical activity (daily energy expenditure), and tobacco smoking status. Factors that were significantly associated with lung function parameters in univariate analyses (*p*-values < 0.1) were considered in the multiple regression models, where the nonsignificant ones at *p* = 0.05 were removed from the final models.

Information on age, sex, ethnicity, and immigration status were collected using a CHMS household questionnaire (Statistics Canada 2010b). Standing height was objectively measured by a fixed stadiometer using standard procedure based on the Canadian Physical Activity, Fitness and Lifestyle Approach (Canadian Society for Exercise Physiology 2003; Statistics Canada 2011). Body mass index (BMI) was calculated using formula weight (kilograms per meter squared). Daily energy expenditure (kilocalories per kilogram per day) was derived from the approach used by the Canadian Fitness and Lifestyle Research Institute (http://www.cflri.ca) and National Population Health Survey (NPHS) (Statistics Canada 2006). Daily energy expenditure (DEE; kilocalories per kilogram per day) was estimated based on the energy expenditure associated with specific activities (MET, the metabolic energy cost associated with specific activity, expressed as kilocalories per kilogram per hour) whose frequency and duration were reported on the CHMS household questionnaire (Statistics Canada 2010b). Information from the CHMS household questionnaire regarding the amount and frequency of cigarette smoking was used to classify each participant as a never, former, or current smoker (Statistics Canada 2010b). Pack-years, defined as number of packs of cigarettes smoked per day multiplied by number of years of smoking, were also calculated using detailed information collected on smoking in the CHMS cycle 1 (Statistics Canada 2010b). In the pack-years calculation, never smokers and former occasional smokers (< 1 cigarette smoked/day in the past) were assigned a value of 0 pack-years.

*Statistical analyses*. Lung function measures FVC, FEV_1_, FEV_1_/FVC, and FEF_25%–75%_ were modeled as continuous health outcome variables in the analyses. In regression analyses, plasma *p,p´*-DDT was dichotomized as detectable (> LOD) or not detectable (≤ LOD = 0.05 μg/L plasma); samples for 90% of participants were ≤ LOD. Plasma *p,p´*-DDE was modeled as a continuous variable because only a small proportion (0.7%) had a concentration ≤ LOD. For participants with *p,p´*-DDE concentrations ≤ LOD, a substitution of 0.5 × LOD was used (Röllin et al. 2009). Chi-square test and Student *t*-test were used to examine the difference in the proportion of detecting *p,p´*-DDT in blood and the mean concentrations of *p,p´*-DDE across demographic factors, respectively.

Design weights provided by Statistics Canada to adjust for poststratification in the multistage sampling, subsampling for the subsurvey, units with no responses, and out-of-scope responses were incorporated in all statistical analyses (Statistics Canada 2011). A resampling method using 500 bootstrap weights was applied to calculate the variance of regression coefficient estimates and 95% confidence intervals (CIs) (Statistics Canada 2011).

Univariate analyses were initially conducted to examine the relationship between risk factors and lung function. Factors that were significant at *p* = 0.1 were considered in the multiple regression models. In multiple regression models, a purposeful selection method was used to determine the final models: The known risk factors of lung function, including age, sex, ethnicity, height and smoking, were forced into the final models, and other variables that were nonsignificant at *p* = 0.05 were excluded from the models.

Associations between lung function parameters and dichotomous *p,p´*-DDT or lipid-normalized *p,p´*-DDE concentrations were estimated by the final multiple linear regression analyses, with lung function as the dependent variable, adjusting for age (continuous), sex, ethnicity (Caucasian or other), height (continuous), smoking status (never, former, current), and daily energy expenditure (continuous). In addition, interactions were not included in final models because none of the interactions between exposures and other covariates on association with lung function outcomes were significant at *p* = 0.05. All statistical analyses were performed with procedures for the complex survey data analysis in STATA (release 12; StataCorp, College Station, TX, USA). This study was approved by the Health Research Ethics Board of the University of Alberta.

## Results

*Characteristics of the study population*. Fasting blood samples for *p,p´*-DDT and *p,p´*-DDE analysis were collected from 1,696 participants 20–79 years of age from five Canadian provinces (Table 1). Among these participants, males and females were almost equally distributed, 22.9% were immigrants, and more than two-thirds had Caucasian ethnicity. The study population had an average height of 169.0 cm and average weight of 77.4 kg. In addition, among this study population, 45.8% never smoked, 31.3% were former smokers, and 22.9% were current smokers.

**Table 1:** Characteristics of the study population.

Characteristic	Percent (95% CI) or mean ± SE
Total sample (*n* = 1,696)
Age [years (%)]
20–39	37.9 (37.9, 37.9)
40–59	41.3 (41.3, 41.3)
60–79	20.7 (20.7, 20.7)
Sex (%)
Female	50.6 (50.4, 50.9)
Male	49.4 (49.1, 49.6)
Height (cm)	169.0 ± 0.4
Weight (kg)	77.4 ± 0.9
Ethnicity (%)
Caucasian	71.4 (62.7, 80.1)
Others	28.6 (19.9, 37.3)
Immigrant (%)
No	77.1 (66.6, 87.6)
Yes	22.9 (12.4, 33.4)
Province of residence (%)
New Brunswick	7.2 (0, 22.1)
Quebec	23.8 (8.9, 38.6)
Ontario	38.9 (38.9, 38.9)
Alberta	16.6 (16.6, 16.6)
British Columbia	13.6 (13.6, 13.6)
Smoking status (%)
Never	45.8 (42.0, 49.6)
Former	31.3 (28.0, 34.6)
Current	22.9 (20.4, 25.4)
Survey design weights were used in calculating percentages and mean values of the study population, a representative sample of the Canadian adults. Survey design weights and 500 bootstrap weights were included in calculating the standard errors (SE) and 95% CIs.	

p,p*´-DDT and* p,p*´-DDE concentrations in the study population*. Of 1,696 participants, 10.0% (95% CI: 4.6, 15.4%) had detectable plasma *p,p´*-DDT (Table 2). A significantly higher proportion of non-Caucasians had detectable *p,p´*-DDT compared with Caucasians (25.6% vs. 3.8%), and immigrants were significantly more likely to have detectable *p,p´*-DDT than nonimmigrants (34.1% vs. 2.9%).

**Table 2:** Plasma *p,p´*-DDT and *p,p´*-DDE among the study population by demographic factors and smoking status.

Characteristic	*p,p´*-DDT		*p,p´*-DDE	
	Percent ≥ LOD (95% CI)	*p*-Value	Mean (95% CI) (ng/g)	*p*-Value
Total sample	10.0 (4.6, 15.4)		326.9 (210.7, 443.0)
Age (years)
20–39	9.1 (3.9, 14.2)		198.6 (115.1, 282.1)
40–59	9.5 (2.6, 16.4)	0.53	281.9 (188.8, 374.9)	0.023
60–79	12.8 (7.4, 18.2)	0.10	648.0 (280.4, 1015.6)	0.014
Sex
Female	11.3 (5.8, 16.8)		418.7 (235.0, 602.5)
Male	8.7 (2.7, 14.8)	0.23	235.4 (169.0, 301.8)	0.021
Ethnicity
Caucasian	3.8 (2.5, 5.1)		197.6 (171.2, 224.1)
Others	25.6 (13.9, 37.3)	< 0.0001	648.4 (305.4, 991.5)	0.015
Immigrant
No	2.9 (1.4, 4.4)		173.9 (153.9, 193.8)
Yes	34.1 (19.5, 48.7)	< 0.0001	650.1 (452.6, 847.7)	< 0.0001
Smoking status
Never	15.3 (6.6, 24.1)		432.4 (217.7, 647.0)
Former	7.1 (2.6, 11.6)	0.056	273.8 (221.6, 326.0)	0.060
Current	3.1 (0.5, 5.7)	0.003	183.0 (141.8, 224.2)	0.033
Mean concentrations of *p,p´*-DDT were below the LOD because a higher proportion of participants had no *p,p´*-DDT detectable in plasma. Mean concentrations of *p,p´*-DDE were calculated among all participants and for participants with concentrations below the LOD (< 1.0%), 0.5 × LOD was used. Survey design weights and 500 bootstrap weights were used in calculating percentages, mean values, and 95% CIs.				

In this study, > 99.0% of participants (95% CI: 99.2, 100) had detectable plasma *p,p´*-DDE (Table 2). The average concentration of *p,p´*-DDE was 326.9 ng/g lipid with a median value of 151.9 ng/g lipid (95% CI: 126.9, 191.8) and an interquartile range of 71.5–284.6 ng/g lipid. On average, females had higher plasma *p,p´*-DDE than males (Table 2). Participants ≥ 60 years of age had a mean concentration of *p,p´*-DDE three times that for participants 20–39 years.

The proportion of participants with detectable *p,p´*-DDT was greater in never smokers than in former and current smokers, and the mean concentration of *p,p´*-DDE was greater in nonsmokers than in current and former smokers (Table 2). In addition, participants with detectable *p,p´*-DDT had a significantly greater mean *p,p´*-DDE concentration compared with those who had no detectable *p,p´*-DDT (1493.3 ng/g lipid; 95% CI: 540.4, 2446.1 vs. 196.1 ng/g lipid; 95% CI: 171.6, 220.6; *p* = 0.012).

*Lung function and detectable plasma* p,p*´-DDT*. After adjusting for age, sex, ethnicity, height, smoking status, and daily energy expenditure, participants with detectable *p,p´*-DDT had a significantly lower mean FVC (difference = 311 mL; 95% CI: –492, –130; *p* = 0.003) and FEV_1_ (difference = 232 mL; 95% CI: –408, –55; *p* = 0.015) than those with nondetectable *p,p´*-DDT (Table 3). *p,p´*-DDT was not associated with the FEV_1_/FVC ratio or FEF_25%–75%_. Model estimates were similar when adjusted for pack-years instead of smoking status (data not shown).

**Table 3:** Results from the multiple linear regression of lung function parameters and plasma *p,p´*-DDT and *p,p´*-DDE

Exposure	FVC (mL)		FEV_1_ (mL)		FEV_1_/FVC (%)		FEF_25–75%_ (mL/sec)	
	β (95% CI)	*p*‑Value	β (95% CI)	*p*‑Value	β (95% CI)	*p*‑Value	β (95% CI)	*p*‑Value
*p,p´*-DDT
< LOD	0		0		0		0
≥ LOD	–310.7 (–491.8, –129.6)	0.003	–231.8 (–408.3, –55.3)	0.015	0.08 (–1.71, 1.87)	0.925	–98.6 (–435.7, 238.5)	0.533
*p,p´*-DDE
per 100 ng/g lipid	–18.8 (–28.7, –8.9)	0.002	–11.8 (–20.6, –3.1)	0.013	0.09 (–0.11, 0.28)	0.363	–2.2 (–27.3, 22.9)	0.850
Beta (β) coefficients were obtained after adjusting for age, sex, ethnicity (Caucasian or other), height, and smoking status (never, former, and current smokers), and daily energy expenditure. Survey design weights and 500 bootstrap weights were included in calculating β coefficients, 95% CIs, and variance estimation.								

*Lung function and lipid-normalized plasma* p,p*´-DDE concentration*. In a multiple linear regression analysis, after adjusting for age, sex, ethnicity, height, smoking status, and daily energy expenditure, each 100-ng/g increase in plasma concentration of *p,p´*-DDE was associated with an 18.8-mL reduction in mean FVC (*p* = 0.002) and an 11.8-mL reduction in mean FEV_1_ (*p* = 0.013) (Table 3). Plasma *p,p´*-DDE was not associated with the FEV_1_/FVC ratio or FEF_25%–75%_. Model estimates were similar when adjusting for pack-years instead of categorical smoking status (data not shown).

## Discussion

DDT was widely used in agriculture and in the control of malaria and typhus before its use was restricted in the 1970s. Although it has been out of use now for many years, the current results from the CHMS cycle 1 (2007–2009) show that almost all Canadian adults 20–79 years of age still had *p,p´*-DDT and/or *p,p´*-DDE detectable in their blood plasma, which is consistent with the data reported by Health Canada using the same survey data (99.6% and 9.3% had detectable plasma *p,p´*-DDE and *p,p´*-DDT, respectively) (Health Canada 2010). In addition, participants who had plasma *p,p´*-DDE concentrations below the LOD also had *p,p´*-DDT nondetectable. Ongoing exposure may arise because of the high persistence of DDT and DDE in the environment (ATSDR 2002). DDT and its metabolites are also highly persistent in the human body, so our results could also be partially or wholly a consequence of exposures some time ago (Longnecker 2005; Wolff et al. 2000). The mean plasma concentration of *p,p´*-DDE reported in this study (152 ng/g lipid adjusted) was lower than that reported from the U.S. National Health And Nutrition Examination Survey (NHANES III, 1999–2004; 238–260 ng/g lipid adjusted) [Centers for Disease Control and Prevention (CDC) 2009; U.S. Environmental Protection Agency (EPA) 2008], indicating a lower exposure to DDT and its related compounds in Canada than in the United States.

Although there have been a number of studies suggesting an adverse effect of pesticides on pulmonary function (Beseler and Stallones 2009; Chakraborty et al. 2009; Fareed et al. 2013; Hernandez et al. 2008; Mekonnen and Agonafir 2004; Peiris-John et al. 2005; Rastogi et al. 1989; Salameh et al. 2005; Zuskin et al. 2008), most have lacked information on the specific types of pesticides used (Beseler and Stallones 2009; Mekonnen and Agonafir 2004; Salameh et al. 2005; Zuskin et al. 2008), whereas others focused on pesticides other than DDT, such as organophosphate or carbamate insecticides (Chakraborty et al. 2009; Fareed et al. 2013; Peiris-John et al. 2005).

We estimated that among a representative sample of Canadian adults 20–79 years of age, participants with detectable plasma *p,p´*-DDT had significantly lower mean FVC and FEV_1_ than those with plasma *p,p´*-DDT ≤ LOD. The estimated magnitude of FVC and FEV_1_ reduction associated with DDT exposure reported in this study (310.7 mL and 231.8 mL, respectively) is similar to the natural decline of lung function (30 mL/year in FVC and 20–30 mL/year in FEV_1_) for healthy nonsmoking adults over 10 years (Burrows et al. 1983; Peat et al. 1990). In addition, lipid-normalized plasma *p,p´*-DDE concentrations were negatively associated with FVC and FEV_1_ when modeled as a continuous variable. To the best of our knowledge, this study is the first population-based investigation of the association of DDT and its metabolite DDE with lung function among Canadian adults.

Several studies in the literature have also reported that exposures to other organochlorine pesticides are associated with reductions in lung function. For example, a study among agricultural pesticide sprayers in Spain reported that exposures to endosulfan were negatively associated with FEV_1_ and FEF_25%–75%_ (Hernandez et al. 2008). Another study among pesticide spraying workers in India reported that a restrictive type of impairment of lung function was associated with exposures to unspecified organochlorine insecticides (Rastogi et al. 1989), which is consistent with the negative association between DDT/DDE and lung function estimated in the present study.

Exposure to DDT has also been associated with the prevalence of respiratory diseases. Hoppin et al. in the Agricultural Health Study reported that DDT exposures were associated with nonatopic asthma among male farmers (Hoppin et al. 2009) and atopic asthma among female farmers (Hoppin et al. 2008). In addition, Hoppin et al. reported that the lifetime number of days of occupational application of DDT in agriculture was significantly associated with higher prevalence of chronic bronchitis (Hoppin et al. 2007). Another study using the same data set found that the prevalence of chronic bronchitis among female nonsmoking farmers was significantly associated with the use of DDT (Valcin et al. 2007).

DDT and related compounds are neurotoxicants that bind to voltage-gated sodium channels to prevent their closure, which leads to increased sodium influx and repeated firing of neurons (Keifer and Firestone 2007). In addition, physiological studies of animal models have shown that sodium influx and subsequent depolarization of neurons in general can cause contractile responses of airway smooth muscles (Souhrada and Souhrada 1989; Souhrada et al. 1988). Moreover, DDT and its metabolite DDE have been shown to be able to activate stress-response signaling *in vitro*, including ERK/MAPK (extracellular-signal-regulated kinase/mitogen-activated protein kinase), JNK (c-Jun N-terminal kinase), and NF-κB (nuclear factor kappa-light-chain-enhancer of activated B cells) signaling pathways, which results in an intracellular release of calcium (Abdollahi et al. 2004; Androutsopoulos et al. 2013). Calcium release into cytoplasm has also been shown to lead to contraction of airway smooth muscles in studies of airway smooth muscle cells and animal model rat studies (Berridge 2008; Menshikova et al. 1995; Sakai et al. 2013; Tomasic et al. 1992). Airway narrowing in response to DDT/DDE exposures would be consistent with the negative associations between exposures and lung function in our study population.

Changes in immune system parameters and markers of immune function have been associated with DDT/DDE exposures in observational studies (Daniel et al. 2001, 2002; Vine et al. 2001), which suggests that exposure might contribute to impaired lung function by increasing airway sensitization or inflammation.

Previous studies of pesticides and respiratory outcomes often have used questionnaire-based approaches or job titles to classify pesticide exposures (Ejigu and Mekonnen 2005; Hernandez et al. 2008; Mekonnen and Agonafir 2004; Salameh et al. 2005; Zuskin et al. 2008), both of which are prone to errors and bias. In this study, we used a biomonitoring approach to measure DDT/DDE exposures, that is, objectively testing the concentration of *p,p´*-DDT/DDE in blood plasma (Kapka-Skrzypczak et al. 2011). The concentration of pesticide measured by biomonitoring method is likely a good estimate of actual body burden arising from exposures to bioaccumulative chemicals, and hence is a good alternative for measuring cumulative exposures. For DDT and DDE, this is particularly so because of their long half-lives in the human body, which makes them a good marker of past or cumulative exposure in research and environmental surveillance projects (CDC 2005; Reigart and Roberts 1999).

There are several limitations in this study. First, the CHMS survey was not fully representative of the Canadian population. Aboriginal people living on reserves and Aboriginal settlements, people living in remote areas, residents of institutions, and full-time members of the Canadian Forces were not included in the CHMS (Statistics Canada 2011). However, the excluded populations in the CHMS represent < 4% of the total Canadian population (Statistics Canada 2011). Second, in the current study, only one of the 13 isomers of the insecticide DDT (Kroschwitz and Howe-Grant 1995), *p,p´*-DDT with its metabolite *p,p´*-DDE, was measured. The rest of the 12 isomers might have been present in blood samples and were not monitored (Statistics Canada 2011). Third, in the present study, associations between DDT/DDE and lung function parameters were characterized among participants 20–79 years of age. Potential effects of DDT and DDE on respiratory health may also be critical for subjects at younger ages. For example, birth cohort studies in Spain have suggested that perinatal exposure to DDT was positively associated with asthma prevalence and persistent wheezing in children (Sunyer et al. 2005, 2006). In addition, associations between respiratory tract infection and DDT/DDE exposures have also been reported among young children (Dallaire et al. 2004; Sunyer et al. 2010). A future study of the effect of DDT/DDE on lung function among children and youth is necessary. Last, due to the cross-sectional nature of the CHMS, the temporal sequence between changes in lung function and DDT exposures is not clear. In addition, because a large proportion of participants had no detectable level of DDT, analyses of DDT using a dichotomous exposure may lead to a potential bias due to uncontrolled confounding or misclassification.

## Conclusions

Although a worldwide ban of DDT for agricultural use has been in place since 2004, when the Stockholm Convention classified it as a persistent organic pollutant, DDT is still currently produced and used in many countries, including China, India, South Africa, Ethiopia, and North Korea (UNEP 2010). Our results show that *p,p´-*DDE, the metabolite of insecticide DDT, was detectable in almost all blood samples of Canadian adults 20–79 years of age, indicating that exposure to DDT is still a health concern, despite a ban in Canada many decades ago. Issues related to the health impact of DDT have been raised since Rachel Carson’s *Silent Spring* was published in the early 1960s (Carson 1962). However, there is still limited evidence for an effect of DDT on respiratory health. Our study is the first population-based study of Canadian adults to demonstrate that plasma DDT and its metabolite DDE were negatively associated with two measures of lung function, specifically FVC and FEV_1_.
