# Erratum: Association between Lung Function in Adults and Plasma DDT and DDE Levels: Results from the Canadian Health Measures Survey

**DOI:** 10.1289/ehp.123-A116

**Published:** 2015-05-01

Ye M, Beach J, Martin JW, Senthilselvan A. 2015. Environ Health Perspect 123:422–427; http://dx.doi.org/10.1289/ehp.1408217Published online 23 December 2014

When this article was originally posted online as an Advance Publication, it included two incorrect units of measure: µg/g lipid (used throughout the text and in Table 2) and mg/g lipid (used in Table 3). The correct unit of measure for all these instances is ng/g lipid. The affected units of measure have been corrected throughout the text and tables.

The authors regret the errors.

